# Effectiveness of 13-Valent Pneumococcal Conjugate Vaccine Against Invasive Disease Caused by Serotype 3 in Children: A Systematic Review and Meta-analysis of Observational Studies

**DOI:** 10.1093/cid/ciy920

**Published:** 2018-10-23

**Authors:** Heather L Sings, Philippe De Wals, Bradford D Gessner, Raul Isturiz, Craig Laferriere, John M McLaughlin, Stephen Pelton, Heinz-Josef Schmitt, Jose A Suaya, Luis Jodar

**Affiliations:** 1 Vaccines Medical Development and Scientific and Clinical Affairs, Pfizer, Inc., Collegeville, Pennsylvania; 2 Department of Social and Preventive Medicine, Laval University, Quebec City, Canada; 3 Vaccines Medical Development and Scientific and Clinical Affairs, Pfizer Canada, Inc., Kirkland, Quebec; 4 Boston University Schools of Medicine and Public Health, Massachusetts; 5 Boston Medical Center, Massachusetts; 6 Vaccines Medical Development and Scientific and Clinical Affairs, Pfizer, Inc., Paris, France; 7 Vaccines Medical Development and Scientific and Clinical Affairs, Pfizer, Inc., New York, New York

**Keywords:** pneumococcal conjugate vaccine, serotype 3, meta-analysis

## Abstract

The 13-valent pneumococcal conjugate vaccine (PCV13) is the only licensed PCV with serotype 3 polysaccharide in its formulation. Postlicensure PCV13 effectiveness studies against serotype 3 invasive pneumococcal disease (IPD) in children have shown inconsistent results.  We performed a systematic review and meta-analysis of observational studies to assess PCV13 vaccine effectiveness (VE) for serotype 3 IPD in children. We systematically searched PubMed, Embase, and the Cochrane library for studies published before 14 August 2017. We identified 4 published studies and 2 conference posters that provided PCV13 VE estimates stratified by serotype. The pooled PCV13 VE against serotype 3 IPD from the random-effects meta-analysis was 63.5% (95% confidence interval [CI], 37.3%–89.7%). A sensitivity analysis including conference posters gave a pooled VE estimate of 72.4% (95% CI, 56.7%–88.0%). The pooled data from case-control studies with similar methodologies and high quality support direct PCV13 protection against serotype 3 IPD in children.

## Teaser

Meta-analysis of published case-control studies of 13-valent pneumococcal conjugate vaccine gave a pooled vaccine effectiveness of 63.5% against serotype 3 invasive pneumococcal disease (IPD). The pooled data from case-control studies with similar methodologies and high quality support direct protection against serotype 3 IPD in children.

*Streptococcus pneumoniae* remains a significant cause of infection associated with high mortality and morbidity in children and adults [1, 2]. A 23-valent pneumococcal polysaccharide vaccine was licensed in 1983 for use only in individuals >2 years of age as it is poorly immunogenic in infants [3]. To overcome these limitations, a 7-valent pneumococcal conjugate vaccine (PCV7, containing serotypes 4/6B/9V/14/18C/19F/23F) was licensed in the United States and European Union in 2000 and 2001, respectively. Ten-valent (PCV10) and 13-valent (PCV13) vaccines were subsequently licensed containing the 7 pneumococcal capsular polysaccharide serotypes in PCV7, plus 3 (1/5/7F [PCV10]) or 6 (1/3/5/6A/7F/19A [PCV13]) additional serotypes that were chosen based on the evolving worldwide epidemiology of pneumococcal disease.

Bacteria of serotype 3 are heavily encapsulated and associated with complicated pneumonias and pneumococcal empyemas worldwide [4]. Currently, PCV13 is the only PCV that contains serotype 3 polysaccharide. PCV13 was licensed based on established immunological correlates of protection and noninferiority comparisons with PCV7, a precursor for which vaccine efficacy was demonstrated [5].

Because there was no prelicensure efficacy trial, estimations of effectiveness against the additional serotypes contained in PCV13 have been obtained from postlicensure observational studies. These studies—conducted with different epidemiological designs, in settings with diverse epidemiological environments and vaccination schedule and uptake—have rendered conflicting results for serotype 3. While some have shown high direct vaccine effectiveness (VE) against serotype 3 invasive pneumococcal disease (IPD) [6], others have shown little or no effectiveness [7]. These conflicting results have led some authors of modeling studies to conclude that PCV13 does not protect against serotype 3 IPD [8, 9]. We therefore conducted a systematic literature review and meta-analysis to assess the direct VE of PCV13 against serotype 3 IPD in infants and children <5 years of age.

## METHODS

An independent group (Optum, Eden Prairie, Minnesota) conducted the literature search in accordance with the Centre for Reviews and Dissemination’s guidance for undertaking reviews in healthcare [10] using the population, interventions, comparators, outcomes, and study design (PICOS) system to define the scope. The research question of interest was larger than the one addressed in the present manuscript, and included VE for all pneumococcal vaccines (including plain polysaccharide and conjugate vaccines) against serogroup 6 and serotype 3 pneumococcal disease in both infants and adults. For this reason, studies published between 1 January 1940 and 14 August 2017 were eligible for inclusion. The search used controlled vocabulary and key words,  limited to English-language articles [11], and was performed in the electronic databases PubMed, Embase, and the Cochrane library (Supplementary Table 1). The current study used only the subset of data on PCV13 VE against serotype 3 IPD.

### Inclusion Criteria to Assess PCV13 VE for Serotype 3 IPD in Children

**Figure 1.**
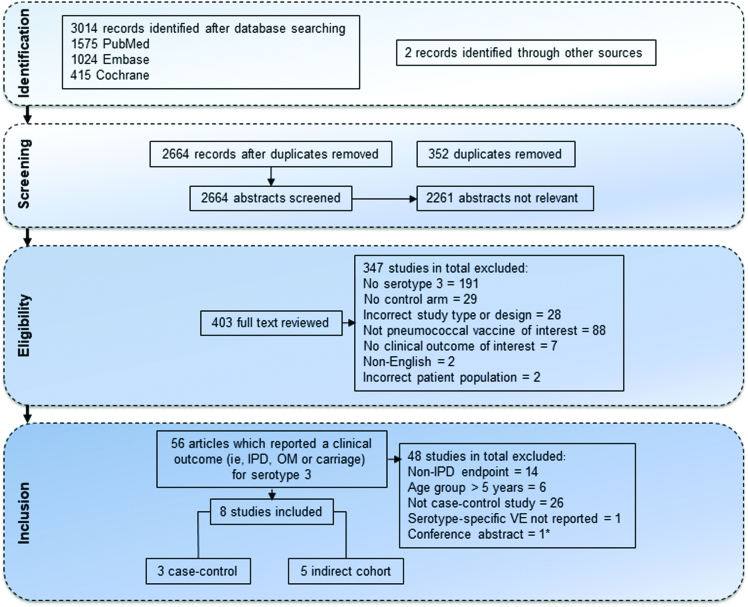
Flowchart of publications included and excluded for this review. Exclusion criteria included studies conducted in nonhuman subjects, with no control arm or reference group (ie, a single-arm study), with incomplete description (usually letters, editorials, or comments), no use of pneumococcal vaccine, or no clinical outcome of interest (eg, serotype-specific antibiotic resistance).  Abbreviations: IPD, invasive pneumococcal disease; OM, otitis media; VE, vaccine effectiveness. *Serotype 3 vaccine effectiveness estimate was later published in study 4.

Two independent reviewers screened titles and abstracts for all potentially relevant publications, based on predefined inclusion and exclusion criteria in accordance with the PICOS method (Figure 1). One independent reviewer was responsible for data extraction, with quality assurance checks performed by a second reviewer.

We included prospective or retrospective observational cohort, case-control, or randomized controlled trials (RCTs) (no RCTs were identified) that provided PCV13 VE estimates stratified by serotype. Studies where children may have received an incomplete vaccination series were included. References within the identified studies were further reviewed for additional pertinent studies.

The study authors who are experts in the field were also aware of 2 relevant posters presented at the European Scientific Conference on Applied Infectious Disease Epidemiology and the International Symposium on Pneumococci and Pneumococcal Diseases. To ensure there was no bias in the identification of these studies, we reviewed the full conference proceedings for these congresses for the period 2015–2018 for relevant studies. We used the Newcastle-Ottawa Scale to assess the quality and bias of the identified published observational studies (Table 1) [12].

**Table 1.** Quality of Published Observational Studies Included in the Review of 13-Valent Pneumococcal Conjugate Vaccine Effectiveness for Serotype 3 Invasive Pneumococcal Disease Using the Newcastle-Ottawa Scale

Study and Location	Study Design	Selection (x/4)	Comparability (x/2)	Exposure (x/3)	Overall (x/9)	Bias^a^
Study 1, United States [6]	Matched case-control	4	2	3	9	Low
Study 2, Spain [13]	Matched case-control	3	2	3	8	Low
Study 3, Germany [14]	Indirect cohort	4	2	2	8	Low
Study 4, United Kingdom [15]	Indirect cohort	4	2	2	8	Low

^a^The scale is categorized into 3 groups: selection, comparability, and outcome/exposure. A maximum of 9 points may be awarded to each study as follows: 4 for selection, 2 for comparability, and 3 for outcome/exposure. Bias was scored as high (0 in any of the categories), moderate (1 in any category), and low (≥2 in all categories).

### Statistical Analysis

Using Stata version 14.0 software (StataCorp LLC, College Station, Texas), we calculated a combined VE estimate using the Stata command Metan to fit a random-effects model [16]. Inverse-variance weighting, which weights each study on the inverse of the variance of each study effect estimate (ie, larger weights for studies with smaller standard errors), was used to combine individual study estimates [17]. This choice of weight minimizes the imprecision of the pooled estimate and is the most commonly used weighting method [18, 19]. To test consistency of the effect estimates across studies, we used Cochran Q-test [20]. We calculated the *I*^2^ statistic to estimate the percentage of between-study heterogeneity [21, 22].

## RESULTS

The initial search identified 3016 publications as potentially relevant (Figure 1). After screening of titles and abstracts, 403 articles were reviewed. Of these, 8 (6 published studies, 2 conference presentations) met the inclusion criteria to assess VE for serotype 3 IPD. However, 2 of the published studies were complete subsets of other identified studies, including 2 studies in Germany (where Weinberger et al [7] provided data that were a complete subset of van der Linden et al [14]) and the United Kingdom (where Miller et al [23] provided data that were a complete subset of Andrews et al [15]). For the meta-analysis, we included only the larger and more recent of these published studies [14, 15].

The 4 published studies were either matched case-control (studies 1 and 2) [6, 13] or indirect cohort studies (studies 3 and 4) (Table 2) [14, 15], 2 of which evaluated VE within a 3 + 1 regimen [6, 14] and 2 within a 2 + 1 regimen [13, 15]. All evaluated effectiveness at least 3 years after PCV13 introduction, included information for both serotype and PCV13 vaccination status, and had low risk of bias (Table 1).

**Table 2.** Description of Case-Control Studies of 13-Valent Pneumococcal Conjugate Vaccine (PCV13) in Children Who Have Reported Data on PCV13 Effectiveness on Serotype 3 Invasive Pneumococcal Disease

Study, Location	Design	Setting/Data Source	Study Period	PCV Use in Infant NIP	Definition/Identification of Cases	Definition/Identification of Controls	Adjustment or Matching of Cases and Controls	Ascertainment of Vaccination Status	Children Immunized With Lower Valent Vaccines Included in VE Analyses for Serotype 3
Published studies									
Study 1, United States [6]	Matched case-control	Population-based IPD surveillance system: ABCs	Jan 2010 to Dec 2014	PCV7: 2000, 3 + 1 PCV13: 2010, 3 + 1	Children with IPD and resident of ABC site	4 controls per case identified via state birth certificate registry	Controls matched for age and location	Medical record	No
Study 2, Spain [13]	Matched case-control	3 pediatric hospitals in Barcelona	Jan 2012 to June 2016	See footnote^a^	Children hospitalized with IPD	4 controls per case; patients admitted to same hospital as cases for cause other than IPD	Controls matched for age, sex, date of hospitalization, and underlying medical condition	Medical record	No
Study 3, Germany [14]	Indirect cohort	Voluntary national IPD surveillance system: German National Reference Center for Streptococci	July 2006 to June 2015	PCV7: 2006, 3 + 1 PCV13: 2009, 3 + 1^b^	Children with IPD reported to National Reference center. Cases = vaccine type IPD	Children with IPD reported to National Reference center. Controls = nonvaccine-type IPD	VE adjusted for age and time period	Questionnaire (diagnostic laboratory)	No
Study 4, United Kingdom [15]	Indirect cohort	National IPD surveillance system: PHE	Apr 2010 to Oct 2013	PCV7: 2006, 2 + 1 PCV13: 2010, 2 + 1	Children with IPD identified by national IPD surveillance system. Cases = vaccine type IPD	Children with IPD identified by national IPD surveillance system. Controls = nonvaccine-type IPD	VE adjusted for age and time period	Questionnaire (general practitioner)	Yes, PCV7
Conference posters									
Study 5, European Union [24]	Indirect cohort	*Streptococcus pneumoniae* invasive disease network	Jan 2012 to Dec 2014	PCV7: 2009–2011, 2 + 1 or 3 + 1; PCV13: 2009–2010 2 + 1 or 3 + 1	Children with IPD identified by active surveillance system. Cases = vaccine type IPD	Children with IPD identified by active surveillance system. Controls = nonvaccine-type IPD	VE adjusted for site, age, sex, underlying conditions, and year of notification	Not reported	Not reported
Study 6, Canada [25]	Unmatched case-control^c^	Children residing in province of Quebec	2005–2016	PCV7: 2002, 2 + 1 or 3 + 1; PCV10: 2009, 2 + 1; PCV13: 2011, 2 + 1	Children with IPD notified to regional public health authority	Children randomly selected in the Quebec Health Insurance Registry	VE adjusted for age, season, calendar year, and presence of high-risk medical conditions	Medical record	PCV7- or PCV10-immunized children considered “not vaccinated”

Abbreviations: ABCs, active bacterial core surveillance; IPD, invasive pneumococcal disease; NIP, National Immunization Program; PCV, pneumococcal conjugate vaccine; PCV7, 7-valent pneumococcal conjugate vaccine; PCV10, 10-valent pneumococcal conjugate vaccine; PCV13, 13-valent pneumococcal conjugate vaccine; PHE, Public Health England; VE, vaccine effectiveness.

^a^The Vaccination Advisory Committee of the Spanish Association of Pediatrics has recommended the routine administration of conjugate pneumococcal vaccines (PCV7 since 2003 to 2010 and, currently, PCV13, 2 + 1). These vaccines were not financed by the Catalan Public Health System until July 2016, except in children with selected risk factors, and were only available in the private sector. In Spain, the vaccine was not introduced into the recommended schedule until January 2017, and there are no estimates of vaccination coverage [13].

^b^PCV13 schedule changed to 2 + 1 in August 2015 except for preterm infants.

^c^Controls were stratifed by age (2–5 months; 6–11 months; and 1, 2, 3, or 4 years of age).

In the matched case-control study in the United States (study 1) [6], VE was estimated as (1 − matched odds ratio) × 100%. Controls were matched for age and location. VE against serotype 3 IPD following ≥1 dose was 79.5% (95% confidence interval [CI], 30.3%–94.8%) (Table 3). These results did not change when adjusted for potential confounders. In the matched case-control study in Spain (study 2) [13], a multivariate analysis was performed using conditional logistic regression that included all demographic, clinical, and epidemiological variables. The adjusted VEs were 25.9% (95% CI, –65.3% to 66.8%) for ≥1 dose; 63.3% (95% CI, –56.2% to 91.4%) for ≥2 doses before 12 months, or 2 doses ≥12 months, or 1 dose ≥24 months; and 12.8% (95% CI, –127.9% to 66.6%) for ≥2 doses before 12 months and 1 dose ≥12 months (Table 3).

**Table 3.** Reported 13-Valent Pneumococcal Conjugate Vaccine Effectiveness for Invasive Pneumococcal Disease

Study, Location	Age	Cases Vacccinated: Unvaccinated^a^	Controls Vaccinated: Unvaccinated^a^	No. of Doses	Serotype 3 VE, % (95% CI)^b^	Reported Range of VE, % (Lowest and Highest) for Other PCV13 Serotypes^c^
Published studies						
Study 1, United States [6]	2–59 m (median, 21–22 m)	16 discordant pairs^d^		At least 1 dose	79.5 (30.3–94.8)^e^	19A: 85.6 (70.6–93.5) 7F: 96.5 (82.7–100)
Study 2, Spain [13]	7–59 m	22:15	91:48	At least 1 dose	25.9 (–65.3 to 66.8)	19A: 86.0 (51.2–99.7) 14: 96.9 (70.4–99.7)
		9:15	22:49	At least 2 doses before 12 m or 2 doses on or after 12 m or 1 dose on or after 24 m	63.3 (–56.2 to 91.4)	The only other serotype with data reported for this schedule was 19A: 85.6 (6.7–99.8)
		12:15	54:49	At least 2 doses before 12 m and 1 dose after 12 m	12.8 (–127.9 to 66.6)	19A: 84.1 (–97.1 to 98.7) 14: 94.2 (41.8–99.4)
Study 3, Germany [14]	74–729 d	6:5	194:43	At least 1 dose	74 (2–93)	19A: 77 (47–90) 6A: 96 (56–100)
	150 to <449 d	1:2	74:20	Postprimary	80 (–68 to 98)	1: 49 (–614 to 96) 7F: 86 (–116 to 100)
	330–729 d	1:2	33:16	Postbooster	63 (–393 to 97)	7F: 32 (–8066 to 99) 19A: 88 (25–99)
Study 4, United Kingdom [15]	4 to ≤56 m	28:21^f^	280:76	At least 2 doses before 12 m or 1 dose on or after 12 m	26 (–69 to 68)	19A: 67 (33–84) 6A: 98 (64–99.8)
	4 to <13 m	3:2	118:20	2 doses before 12 m	66 (–322 to 92)	19A: 62 (–55 to 90) 6A: 96 (41–99.8)
Conference posters						
Study 5, European Union [24]	<5 y	79:50	908:833	At least 1 dose	70 (44–83)^f^	1: 86 (68–93)^g^ 14: 96 (90–100)^g^
		57:34	423:390	Fully vaccinated	57 (5–81)^f^	1: 84 (57–94)^g^ 14: 98 (88–100)^g^
Study 6, Quebec [25]	<5 y	9:27	858:1712	At least 1 dose	20 (–265 to 82)	Not reported

Abbreviations: CI, confidence interval; PCV13, 13-valent pneumococcal conjugate vaccine; VE, vaccine effectiveness.

^a^No. of cases and controls for serotype 3 VE estimates.

^b^If provided, adjusted VE is reported.

^c^Reported range for PCV13 (non–7-valent PCV) serotypes.

^d^The matched odds ratio was calculated using discordant pairs: the number of unvaccinated cases matched to vaccinated controls divided by the number of vaccinated cases matched to unvaccinated controls [26].

^e^Adjusted VE was not reported. However the authors noted the results did not change when adjusted for potential confounders (race, ethnic origin, sex, chronic medical conditions including immunocompromising disorders, low birthweight, exposure to household smoking, daycare attendance, household crowding), recent influenza vaccination (previous 6 months) or influenza disease (previous 30 days), and recent antibiotic use (previous 30 days).

^f^Confidence interval estimated from graph.

^g^Reference 15 reports the number of cases vaccinated vs unvaccinated as 21:28. The correct ratio is 28:21 (Prof Nick Andrews, personal communication).

In the indirect cohort studies, cases of nonvaccine-type IPD were used as controls [27] and VE estimates were adjusted for age and time period. Study 3 from Germany [14] reported VEs of 74% (95% CI, 2% to 93%), 80% (95% CI, –68% to 98%), and 63% (95% CI, –393% to 97%) for ≥1 dose, post–primary series, and postbooster, respectively (Table 3). Study 4 from the United Kingdom [15] reported a VE of 26% (95% CI, –69% to 68%) following ≥2 doses before 12 months or 1 dose ≥12 months of age. VE for ≥2 doses before 12 months was 66% (95% CI, –322% to 92%) (Table 3).

Two of the published studies provided information on the vaccination status of the serotype 3 cases. In study 3 from Germany [14], 11 serotype 3 IPD cases were reported during the 9-year study period, with 5 unvaccinated, 5 incompletely vaccinated, and 1 vaccinated according to schedule. In study 4 from the United Kingdom [15], among 55 serotype 3 IPD cases reported, 22 (40%), 17 (31%), 9 (16%), and 7 (13%) children had received 0, 1, 2, or 3 doses of PCV13, respectively. Though 22 children had received 0 doses of PCV13, for the analysis reporting VE among children with ≥2 doses before age 12 months or 1 dose ≥12 months, 28 children were included as unvaccinated. The reason for considering 6 presumably vaccinated children as unvaccinated was not described.

The authors of the current manuscript identified 2 scientific posters (studies 5 and 6, see Tables 2 and 3) that, though not yet published in peer-reviewed journals, met the inclusion and exclusion criteria and provided complementary evidence beyond the studies described above. Study 5 [24] was conducted by the *Streptococcus pneumoniae* Invasive Disease network (SpIDnet), which is funded by the European Centre for Disease Prevention and Control (ECDC) to perform active population-based surveillance of IPD in children in the European Union [28]. The surveillance system includes >6 million children <5 years of age and data collection across all study sites used a common protocol, allowing for standardization of case definitions, laboratory methods, and approaches for active surveillance [28]. In an indirect cohort analysis of PCV13 effectiveness across all participating PCV13 countries, the adjusted PCV13 VE for serotype 3 IPD was 70% (95% CI, 44%–83%) for ≥1 dose and 57% (95% CI, 5%–81%) for children who were fully vaccinated (Table 3). This study included sites in Spain and the United Kingdom, and thus may have included cases reported in studies 2 and 4.

Study 6 was an unmatched case-control study conducted in Quebec [25]. Thirty-six cases of serotype 3 IPD were reported over an 11-year period (2005–2016) in children 2 months to 4 years of age. Controls were randomly selected from the Quebec Health Insurance Registry and stratified for age. Children were considered as unvaccinated if they had not received PCV13. A total of 9 children with serotype 3 IPD reported between 2006 and 2016 had received ≥1 dose of PCV13, corresponding to an adjusted VE of 20% (95% CI, –265% to 82%) (Table 3).

### Meta-analysis of Vaccine Effectiveness Against Serotype 3 IPD

For the primary meta-analysis, we included the 4 published studies from 4 different countries with nonoverlapping data sets (studies 1–4) [6, 13–15]. For consistency across studies, the estimate for ≥1 dose was used. As study 4 [15] did not report data for ≥1 dose, the lower of the 2 reported VE estimates was used.

**Figure 2.**
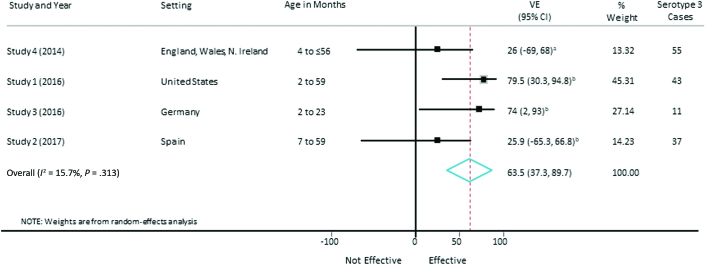
Vaccine effectiveness against serotype 3 invasive pneumococcal disease including only published studies with nonoverlapping datasets. Abbreviations: CI, confidence interval; VE, vaccine effectiveness. ^a^VE for at least 2 doses before 12 months or 1 dose on or after 12 months. ^b^VE for at least 1 dose.

**Figure 3.**
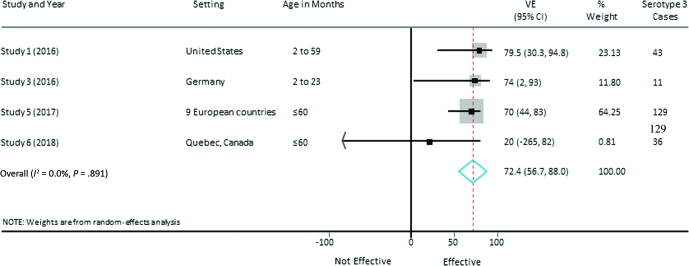
Vaccine effectiveness for at least 1 dose of 13-valent pneumococcal conjugate vaccine against serotype 3 invasive pneumococcal disease including published and unpublished studies with nonoverlapping datasets. Abbreviations: CI, confidence interval; VE, vaccine effectiveness.

The pooled PCV13 VE estimate against serotype 3 IPD from the random-effects meta-analysis was 63.5% (95% CI, 37.3%–89.7%) with low heterogeneity (*I*^2^ = 15.7%, *P* = .313; Figure 2). As a sensitivity analysis, we included both unpublished studies (studies 5 and 6) and excluded the studies from Spain (study 2) and the United Kingdom (study 4), as the cases reported in these studies are included within study 5. In this sensitivity analysis, the pooled VE estimate was 72.4% (95% CI, 56.7%–88.0%) with an *I*^2^ of 0% (*P* = .891; Figure 3).

## DISCUSSION

This is the first study to systematically assess the effectiveness of PCV13 against serotype 3 IPD in children. The pooled data from case-control studies with similar methodologies and high quality support direct PCV13 protection against serotype 3 IPD in children. When the studies were disaggregated, higher VE estimates following ≥1 dose of PCV13 were found in countries with a 3 + 1 vs a 2 + 1 vaccination schedule. However, as the exact vaccination status of the cases and controls in these studies was not reported, it is not possible to determine if a 3 + 1 regimen provides higher protection for serotype 3 IPD than a 2 + 1 regimen. In a RCT in the Netherlands that investigated 4 different PCV13 schedules (two 3 + 1 schedules and two 2 + 1 schedules), there was no significant difference in the serotype 3 geometric mean concentrations postbooster between schedules [29]. In any event, it is difficult to extract conclusions from individual studies. Decisions on the utility of an intervention should rather be based on the totality of the evidence. This is particularly important when the outcome is rare, as is the case for serotype 3 IPD in children, as shown by the small number of reported serotype 3 IPD cases in all of the studies. This emphasizes the value of a meta-analyses of studies with similar methodologies from different sites.

Of the 6 studies identified, 3 used the indirect cohort method [14, 15, 24], which is a variant of the case-control design, with controls being patients with IPD caused by nonvaccine serotypes. This study design has at least 2 methodological advantages. First, as all patients present to the same surveillance center (usually a hospital), it better controls for healthcare system access. Second, to the extent that children with a vaccine type or nonvaccine type have the same opportunity for PCV13 vaccination, and similar risk for the measured outcome, confounding (which requires an association between the hypothesized confounder and both the intervention and outcome) is reduced. However, there are 3 potential disadvantages with this method. If pneumococcal vaccination prevents disease caused by nonvaccine serotypes (eg, PCV13 may provide cross-protection against serotype 6C), VE may be underestimated. Conversely, VE could be overestimated if PCV13 increased risk of nonvaccine-type IPD by increasing the risk of carriage acquisition in children [30]. Finally, with the indirect cohort method, as vaccine coverage increases and vaccine-type disease decreases, the statistical power to demonstrate PCV effectiveness decreases [30].

Our analysis has limitations. First, statistical tests that are used to exclude the hypothesis of a heterogeneity of effect estimates in a meta-analysis (the null hypothesis being the absence of heterogeneity) are not sensitive when the number of studies under review is limited [31]. Second, observational studies are subject to substantial confounding from a variety of biases [32]. This could have affected any of the included studies and could have overestimated VE (if confounding factors resulted in cases being less likely to receive vaccine, eg, because of socioeconomic status) or underestimated VE (if confounding factors resulted in cases being more likely to receive vaccine, eg, children with underlying medical conditions). Two studies conducted multivariable analysis to control for bias [13, 25], but the potential for residual confounding remains. Third, in some studies, many of the cases of serotype 3 IPD were in unimmunized or not properly immunized children, which may have resulted in an underestimation of VE.

The incidence of disease at the population level is the product of both direct and indirect effects, and available surveillance data have shown a low incidence of serotype 3 IPD in children after PCV13 introduction. For example, in the United States, the incidence of serotype 3 IPD among children aged <5 years decreased in the years immediately following PCV13 introduction, dropping from 1.1 cases per 100 000 in 2010 to 0.25 in 2013 [33]. The incidence has since remained low with an incidence of 0.6 in 2016 [33]. In the United Kingdom, based on national surveillance conducted by Public Health England, the number of cases of serotype 3 IPD also declined among children aged <5 years in the years immediately following PCV13 introduction, with a reported incidence rate reduction of 68% (95% CI, 6%–89%) comparing 2013–2014 to 2008 [34]. In the last few years, the number of cases of serotype 3 IPD reported among children aged <5 years has been increasing, but 7 years after PCV13 introduction (in 2016–2017) the incidence of serotype 3 IPD remains lower that that observed before PCV13 introduction [8].

Other findings are important to consider when interpreting data on PCV13 impact against serotype 3. First, PCV13 vaccination leads to a reduction in carriage acquisition and thus indirect protection against vaccine-type disease. PCV13 does not appear to prevent serotype 3 carriage acquisition to the same extent as other vaccine serotypes; however, studies of serotype 3 carriage in children are difficult to interpret due to relatively low carriage prevalence [35, 36]. Nonetheless, the incidence of serotype 3 IPD in unvaccinated age cohorts in countries with PCV13 infant immunization programs has remained relatively constant over time. In contrast, some countries using PCV10 have shown an increase. For example, the ECDC recently reported the indirect effect of childhood PCV vaccination programs in the elderly across 13 sites in the European Union [37, 38]. In sites with universal PCV10 vaccination, the incidence of serotype 3 IPD in adults ≥65 years increased in all sites (pooled increase of 58% in 2015 compared to 2010). In sites with universal PCV13 vaccination, the incidence of serotype 3 IPD in adults showed a pooled decrease of 11%. However, in 2 of the PCV13 countries (Denmark and Norway), there was no change in adult serotype 3 IPD following pediatric PCV13 use [39–41]. In Germany, there has been a significant increase in serotype 3 IPD in adults >60 years of age over the past 3 years [42], again emphasizing the value of analyzing data from multiple geographies.

A coherent hypothesis for PCV13 impact on serotype 3 should take into account all of these data: substantial direct protection; evidence of overall reductions in population-based incidence in the early, but not later, years following introduction; and lower protection afforded by PCV13 against serotype 3 relative to other vaccine serotypes. A recent study has also suggested that there has been a genetic shift from a relatively low to a relatively high antibiotic-resistant serotype 3 clade that was not driven by PCV13 use [43]. One possible explanation that incorporates these observations is that PCV13 provides direct protection against disease and, to a lower extent, carriage. This would explain the initial declines in serotype 3 IPD across all age groups as well as the substantial VE we document in the current manuscript. Subsequently, if a new antibiotic-resistant clade has emerged, at a population level, this antibiotic resistance could lead to increased transmission that reduces, but does not eliminate, the positive benefit of vaccination.

Additional research is needed. First, it is unknown whether the direct protection against serotype 3 is of shorter duration than against other serotypes. Some prelicensure clinical trials showed that the immune response for serotype 3 following the booster dose was not increased above the levels seen after the infant vaccination series, suggesting potential hyporesponsiveness [44]. Despite PCV13 providing direct protection to vaccinated individuals, a combination of lack of impact on carriage and shorter duration of protective immunity may lead to the long-term stagnation or potentially slight increases in overall serotype 3 cases. Second, it will be important to understand whether newly emerging serotype 3 clades represent unique potential vaccine targets, or rather exist on a genotypic and phenotypic continuum. Third, PCV13 is recommended for use in adults (age-based or risk-based recommendations) in 45 countries as of 2016 [3]. VE against adult IPD and nonbacteremic pneumonia due to serotype 3 following direct receipt of PCV13 should also be studied.

The data we present here support protection against serotype 3 IPD from direct vaccination of children with PCV13. This is a first step to understand the full impact of PCV13 in protecting against serotype 3 and the global host, environmental, and pathogen factors that determine serotype 3 epidemiology. This information will be critical for understanding the best public health use of the current vaccine and for developing better vaccines. For example, if PCV13 provides direct protection against serotype 3, but incomplete or no protection against carriage acquisition, it would imply a need for broader reliance on directly immunizing at-risk populations and less reliance on indirect protection from infant immunization programs.

## Supplementary Data

Supplementary materials are available at *Clinical Infectious Diseases* online. Consisting of data provided by the authors to benefit the reader, the posted materials are not copyedited and are the sole responsibility of the authors, so questions or comments should be addressed to the corresponding author.

ciy920_suppl_Supplementary_Table_1

## Notes

***Author contributions.*** All authors participated in the study design, data interpretation, and the writing of the manuscript and agree to be accountable for all aspects of the work.

***Acknowledgments.*** The authors thank Scott Vuocolo, PhD (Pfizer, Inc.), for editorial support. The authors also acknowledge the following individuals from Optum for assistance in conducting the systematic literature review: Laura Pastor, Morgan Kruse, and Michele Kohli oversaw the search and data extraction; David Lanctin and Sarah Rosemas provided support in selecting articles; and Sumit Jhamb, Indu Dhangar, and Dibyajyoti Mazumder did the initial extraction of data from all articles.

***Disclaimer.*** The opinions expressed reflect those of the authors and not necessarily the authors’ institutions.

***Financial support*.** This work was supported by Pfizer, Inc.

***Potential conflicts of interest.*** H. L. S., B. D. G., R. I., C. L., J. M. M., H.-J. S., J. A. S., and L. J. are employees and shareholders of Pfizer, Inc. P. D. W. has received research grants and reimbursements of travel expenses from vaccine manufacturers including GlaxoSmithKline (GSK), Novartis, Sanofi Pasteur, and Pfizer, as well as from governmental agencies including the Quebec Ministry of Health and Social Services, Health Canada, and the Public Health Agency of Canada. Boston Medical Center has received investigator-initiated grants from Pfizer, Inc. and Merck Vaccines with S. P. as principal investigator. S. P. has also received honoraria for participation in advisory board meetings from Pfizer, Inc., GSK Bio, and Seqirus.  All authors have submitted the ICMJE Form for Disclosure of Potential Conflicts of Interest. Conflicts that the editors consider relevant to the content of the manuscript have been disclosed.
