# Corrigendum to: Effectiveness of 13-Valent Pneumococcal Conjugate Vaccine Against Invasive Disease Caused by Serotype 3 in Children: A Systematic Review and Meta-analysis of Observational Studies

**DOI:** 10.1093/cid/ciaa1767

**Published:** 2021-02-12

In the originally published version of this article [Sings HL, De Wals P, Gessner BD, et al. Effectiveness of 13-Valent Pneumococcal Conjugate Vaccine Against Invasive Disease Caused by Serotype 3 in Children: A Systematic Review and Meta-analysis of Observational Studies. Clin Infect Dis 2019;68(12):2135–2143. https://doi.org/10.1093/cid/ciy920], the summary meta-analysis findings were listed as:

63.5% (95% CI, 37.3%–89.7%) with study weights of 45.31, 14.23, 27.14, and 13.32 for studies 1, 2, 3, and 4, respectively, and I^2^ = 15.7% (P = .313; Figure 2) for the main pooled PCV13 vaccine effectiveness (VE) estimate against serotype 3 IPD from the random-effects meta-analysis, and 72.4% (95% CI, 56.7%–88.0%) with study weights of 23.13, 11.80, 64.25, and 0.81 for studies 1, 3, 5, and 6, respectively, and I^2^ of 0% (P = .891; Figure 3) for the random-effects sensitivity analysis that included conference posters.

These estimates should instead be listed as:

50.5% (95% CI, 8.2%–73.3%) with study weights of 17.50, 33.39, 17.06, and 32.05 for studies 1, 2, 3, and 4, respectively, and I^2^ = 32.8% (P = .216; Figure 2) for the main pooled PCV13 VE estimate against serotype 3 IPD from the random-effects meta-analysis, and 69.1% (95% CI, 50.4%–80.8%) with study weights of 13.42, 12.98, 63.62, and 9.98 for studies 1, 3, 5, and 6, respectively, and I^2^ of 0% (P = .574; Figure 3) for the random-effects sensitivity analysis that included conference posters.

The corresponding revised figures are shown below.


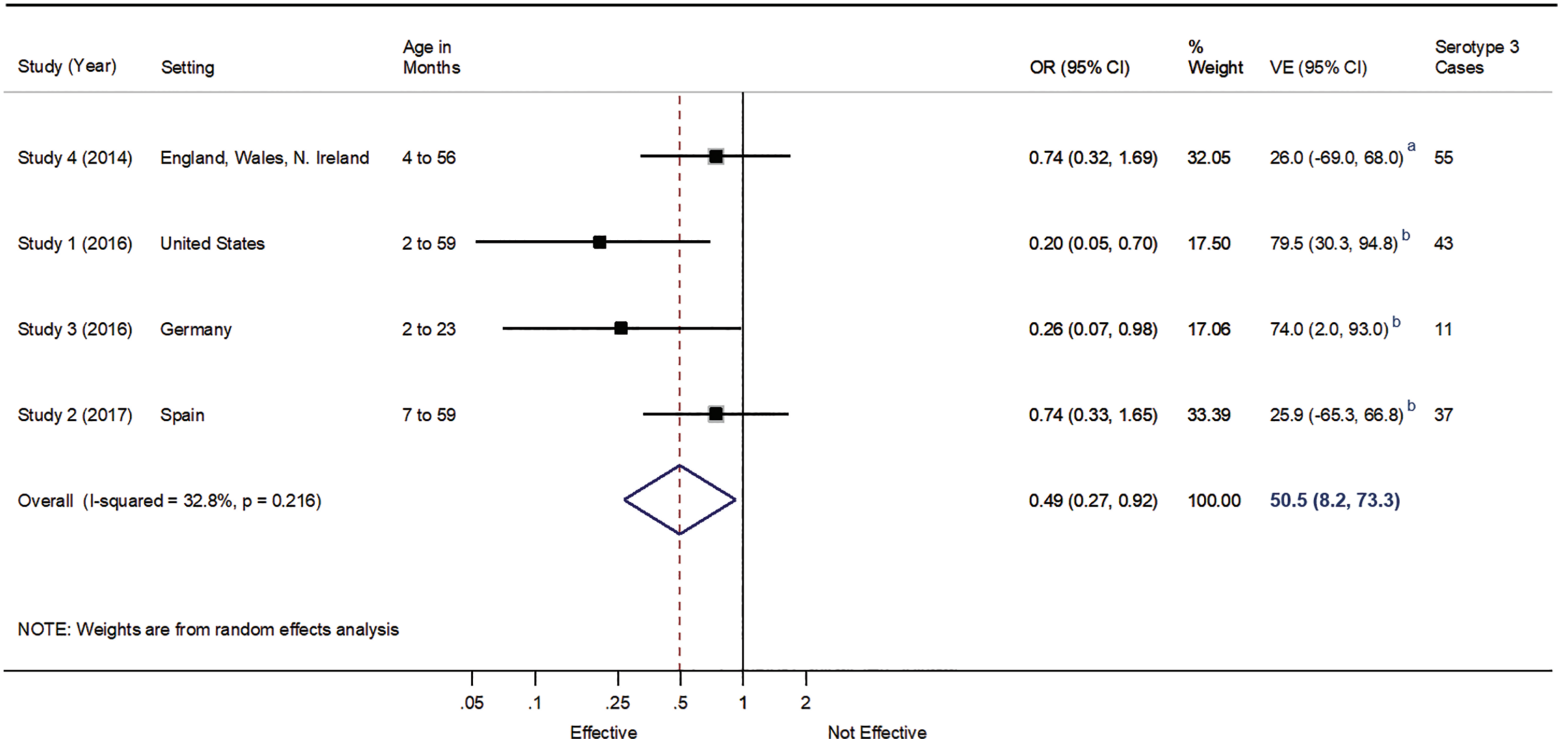


Figure 2 (revised). Vaccine effectiveness against serotype 3 invasive pneumococcal disease including only published studies with nonoverlapping datasets. Abbreviations: CI, Confidence interval; VE, vaccine effectiveness. ^a^VE for at least 2 doses before 12 months or 1 dose on or after 12 months. ^b^VE for at least 1 dose.


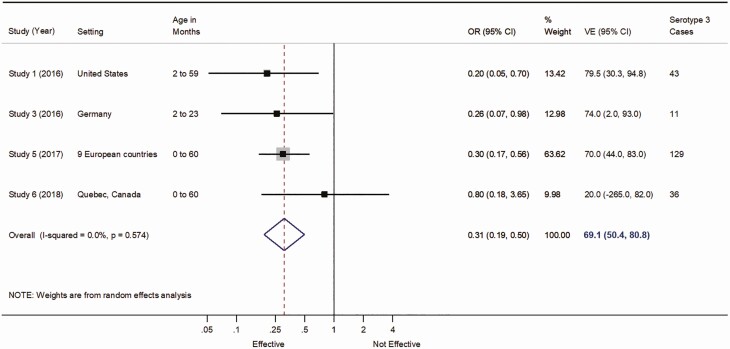


Figure 3 revised. Vaccine effectiveness for at least 1 dose of 13-valent pneumococcal conjugate vaccine against serotype 3 invasive pneumococcal disease including published and unpublished studies with nonoverlapping datasets. Abbreviations: CI, confidence interval; VE, vaccine effectiveness.

The details have been corrected only in this corrigendum to preserve the published version of record. The authors apologize for any inconvenience this may have caused.

