# Supplementary material for: Effectiveness of 13-Valent Pneumococcal Conjugate Vaccine Against Invasive Disease Caused by Serotype 3 in Children: A Systematic Review and Meta-analysis of Observational Studies
Source: Clin Infect Dis. 2018 Oct 23;68(12):2135–43. doi: 10.1093/cid/ciy920 (PMC6541704; doi:10.1093/cid/ciy920)
Supplement: ciy920_suppl_Supplementary_Table_1 [file ciy920_suppl_supplementary_table_1.docx]

Supplementary Table 1. Search strategy.

| **Step** | **Search Criteria** |
| --- | --- |
| 1 | (pneumococc*[Title] OR pneumonia*[Title] OR PCV[Title] OR PPV[Title] OR PPSV[Title]) AND (vaccin*[Title] OR immunis*[Title] OR immuniz*[Title]) |
| 2 | ("Bacterial Vaccines"[Majr]) OR "Pneumococcal Vaccines"[Majr] OR "Streptococcal Vaccines"[Majr] |
| 3 | #1 OR #2 |
| 4 | efficacy[Title/Abstract] OR effect*[Title/Abstract] OR immunogenicity[Title/Abstract] OR opsonophagocytic[Title/Abstract] OR OPA[Title/Abstract] OR antibod*[Title/Abstract] |
| 5 | #3 AND #4 |
| 6 | "Serotyping"[Mesh] OR serotyp*[Title/Abstract] |
| 7 | #5 AND #6 |
| 8 | #7 NOT Filters: Other Animals |
| 9 | #8 Filters: Publication date from 1940/01/01 to 2017/8/14; English |
